# The Sanitary Condition of the London Hospital

**Published:** 1890-04-12

**Authors:** 


### Passing Topics.

### THE SANITARY CONDITION OF THE LONDON HOSPITAL.

The announcement that a house physician of the London Hospital has died "of typhoid fever taken in the discharge of his duties" is a painful comment upon the sanitary condition of this great institution, and a sufficiently serious vindication of the action about to be taken by the authorities to raise its hygienic standard to the level of modern science.

It is doubtless the misfortune rather than the fault of the hospital that it has fallen behind the times in this respect, and that in the performance of its vast and ever-growing work among the sick of the poorest quarter of the metropolis the older portions of the buildings have gradually, perhaps imperceptibly, deteriorated. The London Hospital boasts no advantage of position. Originally erected outside the town, and open to the sweetening influences of breezes fresh from the meadows which lay about its walls when Bow and Stratford were rural villages and Stepney a pleasant riverside resort, it has been surrounded for many a long year by a densely-populated and squalid region where the atmosphere reeks with exhalations from factory, workshop, and crowded house, and, when everything is considered, it is remarkable that the immediate precincts of the hospital should be as wholesome as they are. But whatever the external recommendations, the poisons inevitably generated within the area of a great and crowded hospital are rendered innocuous only by the utmost care and the full use of those advantages the science of sanitation, happily progressive, provides.

It will cause some surprise that the evils consequent upon the ventilation of wards into corridors, the surest means of spreading the contamination arising from less obvious defects, should have escaped notice so long, but it is to the credit of the authorities that they have acted promptly so soon as they have become aware of the danger and have called in to their aid one of the highest sanitary experts of the day. The schedule of requirements which Dr. Louis C. Parkes has submitted to the committee is sufficiently formidable, but when the size of the hospital is considered, it cannot be said that a judicious expenditure of £8,000 or £9,000 upon so paramount an object is excessive. Rather will it appear desirable to most people that this sum should be exceeded by however much is necessary than that one needful improvement should be postponed.

Unfortunately, the London Hospital is poor, and, as we learn by the report, the committee may feel tempted to defer carrying out certain recommendations on merely financial grounds. If this be so, let us say at once that they will scarcely be justified in any such course. Better even that they curtailed their work than that it should be carried on under the grievous and inconsistent condition of sanitary imperfection. The benevolent public, too, may well take note of the position, and by timely help spare the committee the temptation for a parsimony which may mean injury and death to numbers of helpless victims.

In this connection it may be observed that sooner or later there must be a rude awakening for those who think that the work of providing for the treatment of the sick poor can be safely left to be carried on by impecunious institutions. Buildings will continue to deteriorate, and it is more than possible that some are already fit only to be condemned. Under stress of poverty, hospital work is sometimes carried on under conditions which do not afford the best chance of recovery to the patients, and although it might involve a startling proposition to say that the older portions of the London Hospital call for demolition rather than alteration, it is certain that if financial considerations did not possess a dominating influence, we should not be satisfied to lodge one of the greatest and most indispensable of our institutions in a habitation which failed to meet the most exacting requirements of sanitary science. Agricola.

